# Plaque-Associated Oligomeric Amyloid-Beta Drives Early Synaptotoxicity in APP/PS1 Mice Hippocampus: Ultrastructural Pathology Analysis

**DOI:** 10.3389/fnins.2021.752594

**Published:** 2021-11-04

**Authors:** Raquel Sanchez-Varo, Elisabeth Sanchez-Mejias, Juan Jose Fernandez-Valenzuela, Vanessa De Castro, Marina Mejias-Ortega, Angela Gomez-Arboledas, Sebastian Jimenez, Maria Virtudes Sanchez-Mico, Laura Trujillo-Estrada, Ines Moreno-Gonzalez, David Baglietto-Vargas, Marisa Vizuete, Jose Carlos Davila, Javier Vitorica, Antonia Gutierrez

**Affiliations:** ^1^Departamento Biologia Celular, Genetica y Fisiologia, Instituto de Investigacion Biomedica de Malaga-IBIMA, Facultad de Ciencias, Universidad de Málaga, Málaga, Spain; ^2^Centro de Investigación Biomedica en Red Sobre Enfermedades Neurodegenerativas (CIBERNED), Madrid, Spain; ^3^Departamento Fisiologia Humana, Histologia Humana, Anatomia Patologica y Educacion Fisica y Deportiva, Facultad de Medicina, Universidad de Málaga, Málaga, Spain; ^4^Departamento Bioquimica y Biologia Molecular, Facultad de Farmacia, Universidad de Sevilla, Seville, Spain; ^5^Instituto de Biomedicina de Sevilla (IBiS), Hospital Universitario Virgen del Rocio CSIC/Universidad de Sevilla, Seville, Spain; ^6^Department of Neurology, McGovern Medical School, UTHealth Science Center at Houston, Houston, TX, United States

**Keywords:** Alzheimer’s disease, synaptic pathology, hippocampus, transgenic mice (Tg), amyloid, oligomers

## Abstract

Alzheimer’s disease (AD) is a devastating neurodegenerative disorder characterized by initial memory impairments that progress to dementia. In this sense, synaptic dysfunction and loss have been established as the pathological features that best correlate with the typical early cognitive decline in this disease. At the histopathological level, *post mortem* AD brains typically exhibit intraneuronal neurofibrillary tangles (NFTs) along with the accumulation of amyloid-beta (Abeta) peptides in the form of extracellular deposits. Specifically, the oligomeric soluble forms of Abeta are considered the most synaptotoxic species. In addition, neuritic plaques are Abeta deposits surrounded by activated microglia and astroglia cells together with abnormal swellings of neuronal processes named dystrophic neurites. These periplaque aberrant neurites are mostly presynaptic elements and represent the first pathological indicator of synaptic dysfunction. In terms of losing synaptic proteins, the hippocampus is one of the brain regions most affected in AD patients. In this work, we report an early decline in spatial memory, along with hippocampal synaptic changes, in an amyloidogenic APP/PS1 transgenic model. Quantitative electron microscopy revealed a spatial synaptotoxic pattern around neuritic plaques with significant loss of periplaque synaptic terminals, showing rising synapse loss close to the border, especially in larger plaques. Moreover, dystrophic presynapses were filled with autophagic vesicles in detriment of the presynaptic vesicular density, probably interfering with synaptic function at very early synaptopathological disease stages. Electron immunogold labeling showed that the periphery of amyloid plaques, and the associated dystrophic neurites, was enriched in Abeta oligomers supporting an extracellular location of the synaptotoxins. Finally, the incubation of primary neurons with soluble fractions derived from 6-month-old APP/PS1 hippocampus induced significant loss of synaptic proteins, but not neuronal death. Indeed, this preclinical transgenic model could serve to investigate therapies targeted at initial stages of synaptic dysfunction relevant to the prodromal and early AD.

## Introduction

Alzheimer’s disease (AD) is the most common type of dementia, characterized by an initial and gradual memory impairment that eventually affects to other cognitive functions. So far, there is no cure available, and the current treatments are symptomatic with the exception of the recent, and controversially approved, aducanumab (Cummings et al., 2021; Knopman et al., 2021; Mullard, 2021). Considering the growth of elderly population, the number of AD cases worldwide is expected to increase from 50 million today to more than 150 million by 2050 (World Health Organization data, Alzheimer’s Association, 2020). Hence, it is imperative to find new disease modifying therapeutic strategies for this devastating disorder. Accumulation of extracellular amyloid-beta (Aβ) deposits, intracellular formation of neurofibrillary tangles (NFTs), and neuronal loss are the major neuropathological lesions found in AD brains (Hardy and Selkoe, 2002; Long and Holtzman, 2019). Moreover, a recent meta-analysis confirmed that synaptic loss in particular cerebral regions constitutes an early event in AD pathogenesis (de Wilde et al., 2016). In fact, synaptic loss and dysfunction have been considered the best pathological correlation with early cognitive decline (DeKosky and Scheff, 1990; Terry et al., 1991; Selkoe, 2002; Scheff et al., 2007; Arendt, 2009; Shankar and Walsh, 2009; John and Reddy, 2021). In this line, the role played by oligomeric soluble forms of Aβ within the framework of synaptic pathology is becoming increasingly established (Walsh et al., 2002; Shankar et al., 2007; Koffie et al., 2009; revised in Li and Selkoe, 2020). Dystrophic neurites are swollen axons and presynaptic terminals that surround amyloid plaques. These dysfunctional processes are a common AD phenomenon within brain regions related to learning and memory (Su et al., 1993, 1998; Dickson et al., 1999; Dickson and Vickers, 2001), and represent the first event of disease development that might compromise neuronal integrity and synaptic function at the very early stages of AD.

Since 1995, transgenic mice expressing human genes bearing mutations associated with the familiar form of AD (FAD) have been extensively used to unravel the mechanisms underlying synaptic and neuronal degeneration (Forner et al., 2017; Trujillo-Estrada et al., 2021). Most APP-based models usually display plaque-associated dystrophic pathology, loss of synaptic-related proteins, and cognitive deficits, although some results have been contradictory (Webster et al., 2014; Sasaguri et al., 2017). However, these mice generally exhibit scarce or lack of neuronal loss in despite of the accumulation of extracellular/intracellular Aβ aggregates (Wirths and Zampar, 2020).

In this work, we have evaluated the spatial memory and the hippocampal synaptic status of 4.5-month-old APP/PS1 mice. We found an early cognitive decline in hippocampal-related function, in parallel with an altered synaptic profile within this cerebral region, manifested ultrastructurally by a spatiotemporal pattern of synaptic degeneration associated with neuritic plaques and the associated oligomeric-Aβ halo. Importantly, periplaque synaptic damage involved a decrease of synaptic vesicles density (SVD). Thus, dysfunctional presynaptic elements may be a therapeutic target, especially relevant to the prodromal and early stages of this neurodegenerative disease. Finally, this double transgenic mouse model may be considered as a valuable preclinical tool to validate novel treatments targeted at initial phases of synaptic damage.

## Materials and Methods

### Transgenic Mice

Generation and characterization of APP_751*SL*_/PS1_*M*146*L*_ (APP/PS1) mice have been previously reported (Blanchard et al., 2003; Ramos et al., 2006; Caballero et al., 2007; Jimenez et al., 2008, 2011; Moreno-Gonzalez et al., 2009; Baglietto-Vargas et al., 2010, 2017; Sanchez-Varo et al., 2012; Torres et al., 2012; Trujillo-Estrada et al., 2014; Gomez-Arboledas et al., 2018; Sanchez-Mejias et al., 2020). The transgenic mice were obtained by crossing heterozygotic Thy1-APP_751*SL*_ [Swedish (KM670/671NL) and London (V717I) FAD mutations] with homozygous PS1_*M*146*L*_ mice (Charles River, France). Both lines were maintained on a C57BL/6J background. Age-matched wild-type (WT) mice of the same genetic background were used as controls. All animal experiments were performed in accordance with the Spanish and the European Union regulations (RD53/2013 and 2010/63/UE) and approved by the Animal Research Committee from the University of Malaga and Seville (Spain). Experiments and procedures with animals were designed to minimize the animal suffering and reduce the number of animals used.

### Behavioral Studies

The 4.5-month-old mice were submitted to motor function and cognitive tests. Behavioral studies were conducted on age-matched male littermates WT (*n* = 23) and APP/PS1 (*n* = 20). Animals were housed in groups of five and maintained on a 12 h light/dark cycle (lights on at 8.00) at a constant temperature (21 ± 1°C). Behavioral tests were performed during the light period of the light/dark cycle, and the experimenter was blind to the mice genotypes. Habituation and open-field locomotion test (Filali et al., 2012) were performed as described earlier by our group (Trujillo-Estrada et al., 2013; Fernandez-Valenzuela et al., 2020). Monitoring of behavior was done using the software Ethovision XT 7.0 (Noldus, Netherlands).

#### Morris Water Maze Test

This test evaluates spatial cognition and memory using a circular pool (1.4 m diameter, San Diego Instruments, Inc., CA, United States), where animals have to locate a hidden platform beneath opaque water, guided by some spatial cues (Malleret et al., 1999; Hendershott et al., 2016; Fernandez-Valenzuela et al., 2020). Firstly, the mice were habituated to the swimming pool (without the escape platform) for 1 min. The next day, animals performed the visible platform test to discard visual or motivational deficiencies (four trials, maximum trial duration 60 s). During the acquisition phase, mice were trained to find the platform for 4 days (4 trials/day, maximum trial duration 60 s, intertrial interval of 5 min). On the day 5, the platform was removed, and mice were allowed to explore the maze for 60 s to test spatial reference memory (retention phase). Latency, distance traveled, speed, and time spent in the target quadrant (retention phase) were analyzed as described previously (Fernandez-Valenzuela et al., 2020).

### Tissue Preparation

After deep anesthesia with sodium pentobarbital (60 mg/kg), 2-, 4-, 6-, 12-, and 18-month-old APP/PS1 and WT male mice were perfused transcardially with 4% paraformaldehyde, 75 mM lysine, 10 mM sodium metaperiodate in 0.1 M phosphate buffer (PB), pH 7.4. Brains were post-fixed overnight in the same fixative at 4°C, cryoprotected in 30% sucrose, sectioned at 40 μm thickness in the coronal plane on a freezing microtome, and serially collected in wells containing cold PBS and 0.02% sodium azide. Hippocampus-containing sections between bregma coordinates −1.58 and −3.64 mm (Paxinos and Franklin, 2013) were used. For electron microscopy, 4.5-month-old WT and APP/PS1 male mice were perfused transcardially with 0.1 M phosphate buffered saline (PBS)/1% heparin, pH 7.4, followed by 2.5% glutaraldehyde–2% paraformaldehyde in 0.1 M PB, pH 7.4 (Sanchez-Varo et al., 2012). Brains were post-fixed in the same fixative solution overnight at 4°C, washed several times in PB, sectioned at 50 or 100 μm thickness in the coronal plane on a vibratome (Leica VT1000M), and serially collected in wells containing cold PB and 0.02% sodium azide. Then, 100 μm sections were fixed in 2% osmium tetroxide in 0.1 M PB, dehydrated, and embedded in Araldite (EMS, United States). Tissue blocks were serially sliced into semithin (1.5 μm) with a diamond knife in a Leica ultramicrotome (EM UC6), placed on slides, stained with 1% toluidine blue, and scanned under the light microscope for amyloid plaques. Finally, selected areas from semithin samples were cut in ultrathin sections that were placed on Formvar-coated grids and stained with uranyl acetate and lead citrate before being examined with an electron microscope (JEOL JEM 1400). In addition, fixed brains from 6-month-old mice were selected to be washed in PBS 0.1 M and directly sectioned at 50 or 250 μm in the coronal plane on a vibratome (Leica VT1000M). Then, semithin sections (1 μm thickness) were serially collected from embedded tissue blocks using a Leica ultramicrotome (EM UC6) and mounted on gelatine-coated slides. Hippocampus-containing sections between bregma coordinates −1.58 and −3.64 mm (Paxinos and Franklin, 2013) were used.

### Human Brain Samples

Human AD brain tissue samples from medial temporal lobe (hippocampal/parahippocampal regions) were obtained from the Institute of Neuropathology Brain Bank IDIBELL-Hospital Universitari de Bellvitge (Barcelona, Spain) at autopsy (*n* = 5, Braak V–VI stage, and clinically classified as demented AD cases) with a *post mortem* delay between 3 and 16 h (Supplementary Table 1). The utilization of brain samples was approved by the corresponding ethics committees following Spanish legislation. Samples of the hippocampal cortex were fixed in 4% paraformaldehyde in 0.1 M PB for 24–48 h, cryoprotected in 30% sucrose, stored at −80°C, sectioned (30 μm thickness) on a freezing microtome, and serially collected in wells containing 0.1 M phosphate buffer saline (PBS) and 0.02% sodium azide. Following a neuropathological examination, AD stage was defined according to Braak and Braak (1991). Brain samples were used for light microscopy studies.

### Antibodies

The following primary antibodies were used for immunohistochemistry: anti-human amyloid precursor protein (hAPP) rabbit polyclonal antibody (1:20,000, Sigma-Aldrich), anti-amyloid oligomers (OC) rabbit polyclonal (1:5,000, Millipore), anti-Aβ mouse monoclonal 6E10 (1:1,500, Sigma-Aldrich), anti-Aβ42 rabbit polyclonal (1:40, Biosource 44-344), anti-microtubule-associated protein 1 light chain 3 (LC3b) goat polyclonal (1:1,000, Santa Cruz Biotechnology), anti-MAP2 rabbit polyclonal (1:5,000, Chemicon), monoclonal anti-oligomeric Aβ antibody NU-4 (1:2,000; kind gift from Dr. Klein; Northwestern University, IL, United States; Lambert et al., 2007), anti-PSD95 rabbit polyclonal (1:1,000, Invitrogen), anti-synaptophysin (SYN) rabbit polyclonal (1:1000, Abcam), and anti-VGLUT1 polyclonal guinea-pig (1:10,000, Chemicon). The following antibodies were used for molecular studies: anti-beta actin mouse monoclonal (1:5,000, Sigma-Aldrich), anti-NeuN mouse monoclonal (1:1,000, Chemicon International), anti-PSD95 rabbit polyclonal (1:1,000, Cell Signaling), anti-Synaptophysin rabbit polyclonal (1:1,000, Synaptic Systems), anti-VGLUT1 rabbit polyclonal (1:2,000, Synaptic Systems), and anti-VGAT rabbit polyclonal (1:5,000, Synaptic Systems).

### Light Microscopy Immunohistochemistry

The immunolabeling procedures were done as described earlier (Sanchez-Varo et al., 2012). Serial sections were processed in parallel using the same batches of solutions to minimize variability during immunostaining processing. Free-floating sections were first treated with 3% H_2_O_2_/10% methanol in PBS, pH 7.4, for 20 min to inhibit endogenous peroxidases, and with avidin-biotin Blocking Kit (Vector Labs) for 30 min to block endogenous avidin, biotin, and biotin-binding proteins. For single immunolabeling, sections were incubated with one of the primary antibodies over 24 or 48 h at room temperature. The tissue-bound primary antibody was then detected by incubating for 1 h with the corresponding biotinylated secondary antibody (1:500 dilution, Vector Laboratories), followed by streptavidin-conjugated horseradish peroxidase (90 min, 1:2,000, Sigma–Aldrich). The peroxidase reaction was visualized with 0.05% 3-3-diaminobenzidine tetrahydrochloride (DAB, Sigma–Aldrich), 0.03% nickel ammonium sulfate, and 0.01% hydrogen peroxide in PBS. Some sections immunolabeled for SYN were incubated 3 min in a solution of 20% of Congo red. Immunohistochemistry in semithin sections was performed as follows: 50-μm-thick sections were dehydrated, flat embedded in Araldite, and sectioned at 1–1.5 μm thickness using an ultramicrotome (Leica VT1000M). After etching the resin, mounted sections were immunolabeled for OC using the same procedure described before, and then were counterstained with Harry’s hematoxylin (Panreac).

Double immunoperoxidase MAP2/6E10 labeling was performed as described previously (Moreno-Gonzalez et al., 2009). Sections were first incubated in anti-MAP2, and after the DAB-nickel reaction (dark blue end product), sections were incubated in 6E10 antibody. The second immunoperoxidase reaction was develop with DAB only (brown reaction end product). Sections were mounted on gelatine-coated slides, air dried, dehydrated in graded ethanol, cleared in xylene, and coverslipped with DPX (BDH) mounting medium. Specificity of the immune reactions was controlled by omitting the primary antisera.

For double immunofluorescence labeling, sections were first sequentially incubated with the indicated primaries antibodies followed by the corresponding Alexa488/568 secondary antibodies (1:1,000; Invitrogen). OC-immunolabeled sections were stained with 0.02% Thioflavin-S (Sigma-Aldrich) in 50% ethanol. Human sections were previously incubated in autofluorescence eliminator reagent (Millipore), following the manufacturer’s recommendations, to eliminate fluorescence emitted by intracellular lipofuscin accumulation. Finally, all sections were coverslipped with 0.01 M PBS containing 50% glycerine and 3% triethylenediamine and examined under a confocal laser microscope (Leica SP5 II).

### Electron Microscopy Immunogold Labeling

The immunogold procedure was done as we previously described (Sanchez-Varo et al., 2012; Gomez-Arboledas et al., 2018). Sections (50 μm) from 4.5- and 6-month-old APP/PS1 mice were first washed in PBS and incubated in a 50 mM glycine solution 10 min, or cryoprotected in a 25% sucrose and 10% glycerol solution, followed by freezing at −80°C, to increase the antibody-binding efficiency. After following the standard protocol, the tissue was incubated overnight in primary anti-oligomeric OC rabbit polyclonal (1:5,000, Millipore), mouse monoclonal anti-oligomeric Aβ (12–24 mers) NU-4 (1:2,000), or anti-Aβ42 rabbit polyclonal (1:5,000, Abcam) antibodies in a PBS 0.1 M/1% BSA solution at room temperature. Then, sections were washed in PBS and incubated with 1.4 nm gold-conjugated secondary antibody (1:100, Nanoprobes) overnight at room temperature. After post-fixing with 1% glutaraldehyde and washing with 50 mM sodium citrate, the labeling was enhanced with the HQ Silver^TM^ Kit (Nanoprobes). Finally, the immunolabeled sections were processed as we previously described by the osmium fixation, dehydration, and embedding steps. The primary antibody was omitted in negative control experiments.

### Plaque Loading

Plaque loading was defined as the percentage of total hippocampal area stained with the anti-Aβ mouse monoclonal antibody 6E10 (1:1,500, Sigma). Quantification of extracellular Aβ content was performed similarly to the previously reported method (Moreno-Gonzalez et al., 2009; Baglietto-Vargas et al., 2010, 2017; Trujillo-Estrada et al., 2014). Sections were examined under a Nikon Eclipse 80i microscope, and images were acquired with a Nikon DS-5M high resolution digital camera using the ACT-2U imaging software (Nikon Corporation, Minato, Tokyo, Japan). The camera settings were adjusted at the start of the experiment and maintained for uniformity. Digital 4× images (2560 × 1920, 300 ppp) from 2- to 18-month-old APP/PS1 mice (5 sections/mouse; *n* = 4 mice/age) were analyzed using Visilog 6.3 analysis program (Noesis, France). The hippocampal area in each image was manually outlined. Then, 6E10-positive plaque area within the hippocampus was identified by level threshold which was maintained throughout the experiment for uniformity. The color images were converted to binary images with plaques. The plaque loading (%) for each transgenic mouse was estimated and defined as (sum plaque area measured/sum hippocampal area analyzed) × 100. The sums were taken over all slides sampled, and a single plaque burden was computed for each mouse. The mean and standard deviation (SD) of the plaque loading were determined using all the available data (mean hippocampal area analyzed/animal 1901893 ± 80315.51 μm^2^; 618430.92 ± 42359.93 μm^2^ for CA1 region). Quantitative comparisons were carried out on sections processed at the same time with same batches of solutions.

### Synaptophysin Non-immunoreactive Areas and Plaque Size Morphometric Analysis

The quantification of synaptophysin-immunonegative area (SIA) was performed over hippocampal sections (between −0.94 mm anterior and 3.64 mm posterior to Bregma) from 2- to 18-month-old APP/PS1 stained with anti-SYN (*n* = 4 mice/age). The SIA analysis was done using the nucleator method with isotropic probes (*n* = 5 radii) over 992 immunonegative areas by the NewCast software (Olympus). The different hippocampal layers were manually outlined at low magnifications [*stratum oriens* (SO), *stratum radiatum* (SR), and *stratum lacunosum moleculare* (SLM) from CA1, *stratum lucidum* (SL) from CA3, and *stratum moleculare* (SM) from dentate gyrus (DG)] following the atlas of Paxinos and Franklin (2013) to establish the corresponding boundaries. Then, they were analyzed using a counting frame of 8840.7 and 7072.6 μm^2^, respectively, and a step length of 194.02 × 194.02 μm. Individual immunonegative-area measurements were performed under a 40×/0.95 objective; then, the summation was calculated for each layer. SIA was defined as the percentage of the non-immunoreactive area related to the total surface analyzed. To estimate plaque size, digital panoramic ultramicrophotographs (JEOL JEM 1400) of extracellular deposits (*n* = 15) were analyzed similarly to SIA estimation, using the nucleator method with isotropic probes (*n* = 5 radii).

### Synapse and Synaptic Vesicle Quantification

Hippocampal ultrathin sections (60–70 nm) with evident amyloid plaques were selected among serial semithins sections from 4.5-month-old APP/PS1 mice (*n* = 3). Taking the plaque border (defined as the point from which there were no detectable amyloid fibers extending from the deposits in the cerebral parenchyma) as a point of reference, six consecutive ultramicrophotographs were captured in 3–4 different radii (step length ∼4.7 μm; 21.7 μm^2^/image). Moreover, 30 ultramicrophotographs/animal were captured in areas far from plaques (at least, 70 μm distance) to be used as control interplaque regions. For WT mice, three starting points were randomly selected at low magnifications (4,000×) from each analyzed region (SO, SM, and SR). Then, 10 consecutive ultramicrophotographs were captured following the same parameters defined for transgenic mice. Thus, 30 ultramicrophotographs (3 × 10) were taken from each region/animal. Sampling was thoroughly performed to avoid overlapping images (DeFelipe et al., 1999). The density of synaptic boutons within each periplaque (distance of 0–5, 5–10, 10–15, 15–20, 20–25, and 25–30 μm) or free-of-plaque regions (either interplaque or control areas) was estimated considering only those synapses displaying a visible presynaptic element (with more than three synaptic vesicles), the synaptic cleft, and a post-synaptic density. Then, using the same digital images, the SVD measurement was undergone over the synaptic boutons showing a minimum section surface of 0.5 μm^2^ and counted in the area that included the high electron dense postsynaptic zone, as it has been previously published by others (Parodi et al., 2010). For this assay, we established three range of distances from plaque border (0–10, 10–20, and 20–30 μm). Each analysis was done by a single examiner blinded to sample identities.

### Treatment of Primary Neuronal Cultures With Soluble S1 Hippocampal Fractions

The S1 fractions were prepared as described previously (Jimenez et al., 2008; Sanchez-Mico et al., 2021). Primary neuronal cultures were done essentially as described by Jimenez et al. (2011). Briefly, embryonic E18–20 or postnatal P1 brains were dissected and treated, for 5 min, with trypsin-DMEM-EDTA medium (Biowhittaker, Cambrex, Belgium). The treatment was stopped using complete DMEM plus 10% FBS and the cells were mechanically dissociated. Then, the debris were eliminated by filtration (40 μm, BD Falcon) and the cells were cultured (at a density of 60,000 cells/ml) in Neurobasal medium plus B27 supplemental (containing glutamine, 1% penicillin-streptomycin, and gentamycin) on poly-D-lysine (Sigma-Aldrich) treated Nunc 12-well plates. The cells were cultured at 37°C in humidified 5% CO_2_/95% atmosphere. Medium was half-replaced every 4 days. After 13–15 days in culture, the cultures were treated with 10 μg of S1 protein/100 μl of medium. The cells were incubated for 24 h and rinsed with PBS, and the proteins were extracted (see below).

### Total Protein Extraction and Western Blots

Hippocampal samples from 2-, 4-, and 6-month-old APP/PS1 and WT male mice were used (mean sample size 5/group: APP/PS1 mice *n* = 6/age, WT *n* = 4/age). The protein pellets, obtained using the Tripure Isolation Reagent and isopropanol-mediated precipitation, were resuspended in 4% SDS and 8 M urea in 40 mM Tris–HCl, pH 7.4, and rotated overnight at room temperature to get complete protein solubilization (Ramos et al., 2006). Western blots were performed as described (Araujo et al., 1996). Briefly, 0.5–15 μg of proteins from the different samples were loaded on 10%-SDS-tris-glycine-PAGE and transferred to nitrocellulose (Hybond-C Extra; Amersham). After blocking, using 5% non-fat milk TPBS, membranes were incubated overnight, at 4°C, with the appropriate primary antibody. Membranes were then incubated with the corresponding horseradish-peroxidase-conjugated secondary antibody (Dako, Denmark) at a dilution of 1:10,000. Each blot was developed using the ECL-plus detection method (Amersham) and quantified using ImageQuant Las 4,000 mini gold (GE Healthcare Bio-Sciences). For normalization purposes, proteins were first estimated by Lowry and protein loading corrected by beta-actin.

### Statistical Analysis

Data were expressed as mean ± SD. Cognitive data were expressed as mean ± SEM. Parametric and non-parametric statistical analyses were used when appropriate. The comparison between two groups was done by two-tailed *t*-test. To compare several groups, we used one-way ANOVA, repeated-measures (RM) one-way and two-way ANOVA tests, or Kruskal–Wallis, followed by Tukey’s *post hoc* multiple comparison test or Dunn test, respectively. The significance was set at 95% of confidence. Statistical analysis was performed using the GraphPad 7.0 (Prism) software.

## Results

### APP/PS1 Mice Display Early Spatial Memory Deficits

**FIGURE 1:**
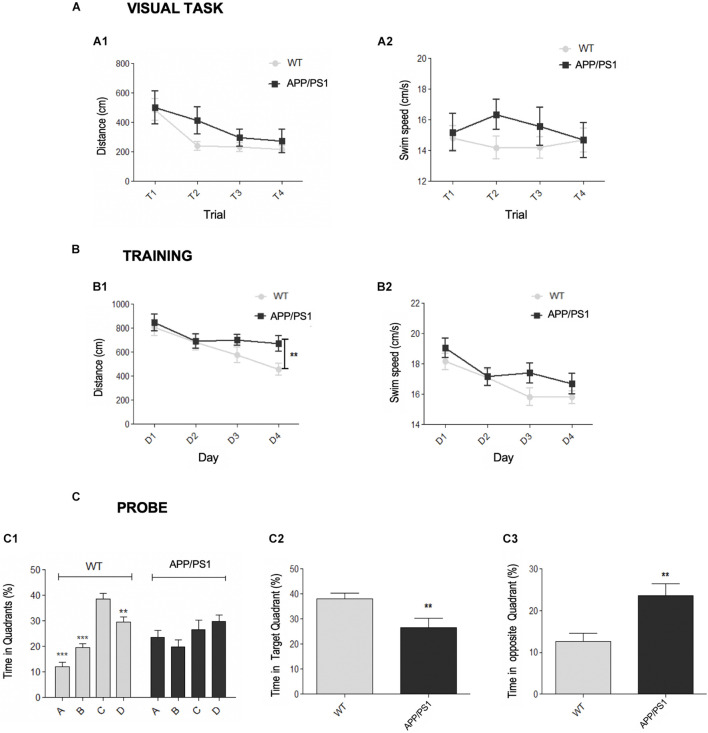
Early spatial memory deficits in APP/PS1 model. **(A–C)** Morris Water Maze (MWM) test for APP/PS1 and WT 4.5-month-old mice. **(A)** Visual task. Two-way RM ANOVA analysis demonstrated that both genotypes correctly performed the visual task (escape latencies: [*F*_(1_, _123)_ = 0.26, *p* = 0.6152]; distance swum: [*F*_(1_, _123)_ = 1.74, *p* = 0.1946]). Differences in both latency [*F*_(1_, _123)_ = 7.06, *p* = 0.0002] and distance traveled [**A1**, *F*_(3_, _123)_ = 7.06, *p* = 0.0002] were found across the trials, demonstrating the improvement along the task-course. No significant interaction effect was found on either latency [*F*_(3_, _123)_ = 1.68, *p* = 0.1752] or distance [*F*_(3_, _123)_ = 0.62, *p* = 0.6037]. **(A2)** Respect to the swim speed no genotype [*F*_(1_, _123)_ = 1.12, *p* = 0.2965], trial [*F*_(3_, _123)_ = 0.18, *p* = 0.9069] or interaction [*F*_(3_, _123)_ = 0.76, *p* = 0.5203] effects were found. **(B)** Acquisition training. Two-way RM ANOVA (training days × genotype) over the training days did not reveal significant differences between the genotypes in either distance [**B1**, *F*_(1_, _123)_ = 2.54, *p* = 0.1189] or swim speed [**B2**, *F*_(1_, _123)_ = 1.83, *p* = 0.1838], neither interaction effect in distance [*F*_(3_, _123)_ = 1.70, *p* = 0.1716] nor velocity [*F*_(3_, _123)_ = 1.01, *p* = 0.3387]. Student’s *t*-test analysis demonstrated that APP/PS1 traveled more distance to reach the platform in the last training day (**B1**, D4: **p = 0.0095) than control group, without significant differences in velocity (**B2**, *p* = 0.3243). **(C)** Probe trial. **(C1)** One-way RM ANOVA intra-group analysis showed significant differences among the quadrants [*F*_(3, 28)_ = 2.24, *p* < 0.0001] for WT group. *Post hoc* analysis showed that control mice spent more time in the target *(C)* than in the adjacent *(B,D)* or opposite *(A)* quadrants (**p < 0.01, ***p < 0.001). In contrast, there were no significant differences in the time spent by the APP/PS1 mice in any quadrant (*p* = 0.2116). *T*-test intergroup analysis showed significant differences between the genotypes in the time spent in both the target quadrant *C* (**C2**, **p < 0.01) and the opposite quadrant *A* (**C3**, **p < 0.01).

First, the motor and cognitive status of young (4.5-month-old) APP/PS1 mice were assessed. Spatial memory was checked using the Morris water maze (MWM) test, a hippocampal-dependent task (Morris, 1984). Previously, the presence of visual or motivational defects was discarded with the visible platform task (Figure 1A). This test was successfully performed by both genotypes, showing differences across the training trials in both latency (time to reach the platform) and distance traveled. Thus, these results demonstrated that both WT and APP/PS1 mice were able to find the platform, progressively diminishing the time or distance to reach it (Figure 1A1). Respect to the swim speed (Figure 1A2), no genotype, trial, or interaction effects were found.

During acquisition phase, mice learned to locate the hidden platform by spatial navigation, as demonstrated by the progressive decrease in the distance traveled along training days. Statistical analysis (Figure 1B) did not reveal a genotype effect, since there were no significant differences in either distance (Figure 1B1) or swim speed (Figure 1B2) between groups, neither interaction effect in distance or velocity. The distance traveled [*F*_(3, 123)_ = 9.72, *p* < 0.0001) and the swim speed (*F*_(3, 123)_ = 11.48, *p* < 0.0001] were changing along the course of the training. Importantly, the last training day (D4), there were significant differences between WT and APP/PS1 groups in the traveled distance to reach the platform, without differences in swim speed. Therefore, APP/PS1 mice spent more time to reach the target, indicating learning impairment.

During the probe trial (Figure 1C), significant differences were detected regarding the time spent within the quadrants by WT or APP/PS1 mice (Figure 1C1): control group stayed longer in the target zone (quadrant C) than in the opposite (A) or adjacent (B, D) quadrants. By contrast, intra-group APP/PS1 analysis did not yield statistically significant differences among the four quadrants. In addition, APP/PS1 mice spent significantly less time in the target quadrant than in the others (C, Figure 1C2), and more time within the quadrant A, in comparison to control group (Figure 1C3). Overall, these data indicated that double transgenic mice displayed memory deficiencies since early ages.

### Early Loss of Synaptic Markers in the Hippocampus of APP/PS1 Mice Progresses in Parallel to Amyloid-Beta Accumulation

**FIGURE 2:**
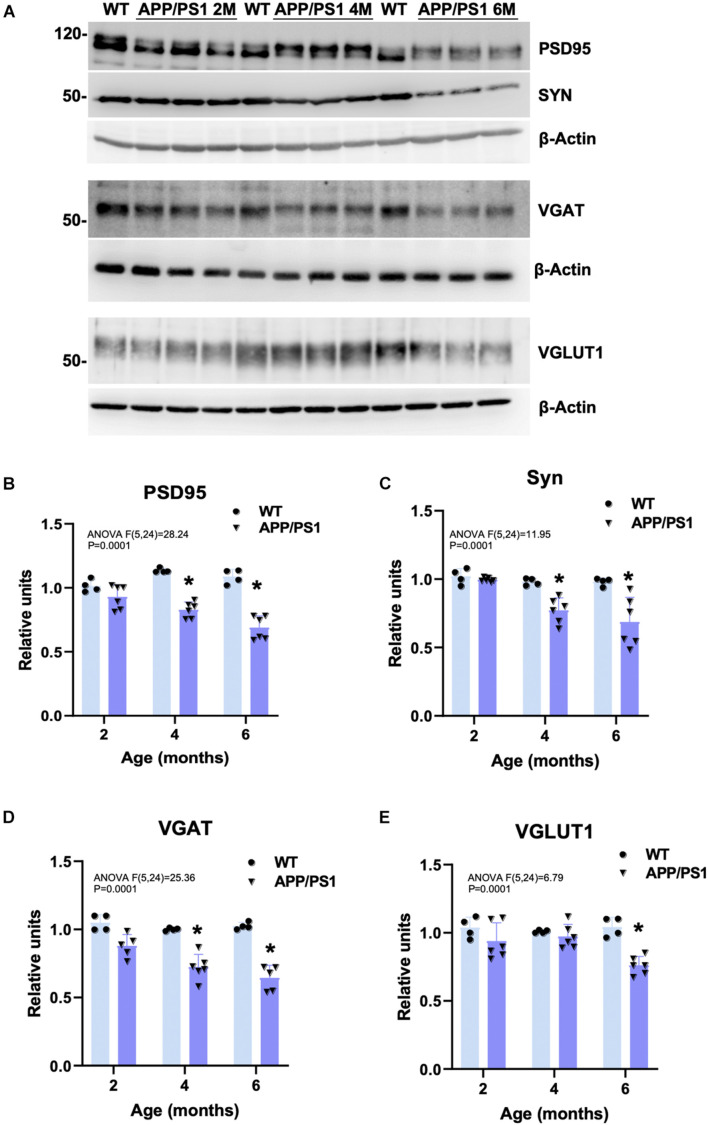
Decreased hippocampal synaptic markers in young APP/PS1 mice. **(A)** Representative Western blots for several synaptic markers in 2-, 4-, and 6-month-old APP/PS1 (*n* = 6/age) and WT (*n* = 4/age) hippocampal samples. Quantitative analysis showed a significant and progressive decrease in the postsynaptic marker PSD95 **(B)**, and the presynaptic markers SYN **(C)** and VGAT **(D)** from 4 to 6 months of age in the double transgenic mice compared to age-matched WT. Moreover, levels of VGLUT1 were significantly reduced at 6 months of age **(E)**. Represented data were normalized respect to 2-month-old WT samples. Tukey’s test: *p < 0.05.

To check whether the detected cognitive deficits correlate with an early synaptic dysfunction, the amount of synaptic proteins was measured by Western blots in hippocampal protein extracts obtained from 2-, 4-, and 6-month-old APP/PS1 and WT mice (representative blots are shown in Figure 2A). Levels of the postsynaptic marker PSD95 (Figure 2B), and the presynaptic synaptophysin (SYN; Figure 2C), and the GABA vesicular transporter VGAT (Figure 2D) were already significantly lower in APP/PS1 than in WT animals at 4 months of age (−27.04 ± 6.09%, −21.29 ± 9.12%, and −27.68 ± 9.31%, respectively). Moreover, the level of these synaptic markers was even more reduced at 6 months (−36.66 ± 9.17%, −29.97 ± 17.97%, and −37.12 ± 9.15%, respectively), along with a decrease in the vesicular glutamate transporter 1 (VGLUT1; −26.94 ± 6.53%; Figure 2E), only detected from 6 months. In addition, SYN- and VGLUT1-immunostainings demonstrated that the presynaptic terminals were directly affected by the presence of Aβ deposits from early ages (Supplementary Figures 1A–D,G,H,M–P). SYN immunoreactivity was virtually absent in some neuropil areas, which appeared to correspond with the location of amyloid plaques, as confirmed by both Congo red staining (Supplementary Figures 1E,F). Similarly, MAP2 (dendrites) and PSD95 (dendritic spines) immunostainings showed the loss of postsynaptic elements because of the plaques (Supplementary Figures 1I–L, double MAP2/6E10 labeling evidence plaque impact in J). All these data together with the ultrastructural inspection using transmission electron microscopy (TEM) indicate that the areas of brain parenchyma occupied by fibrillary plaques were devoid of synaptic contacts. Therefore, the memory impairment observed in young APP/PS1 mice correlates with a reduction of critical synaptic proteins. Our results suggest that this decrease in synaptic markers may be (at least partly) due to extracellular amyloid deposition (hippocampal plaque loading in APP/PS1 with 6E10 antibody: 0 ± 14.26% at 2 m; 5.14 ± 5.7% at 4 m; 7.15 ± 3.65% at 6 m; 13.73 ± 4.9% at 12 m, and 21.35 ± 4.92% at 18 m; see graphics from Supplementary Figures 1M–Q).

### Amyloid Plaques Are Associated With Degenerating Synaptic Boutons Containing Autophagic Vesicles, APP, and Amyloid-Beta

**FIGURE 3:**
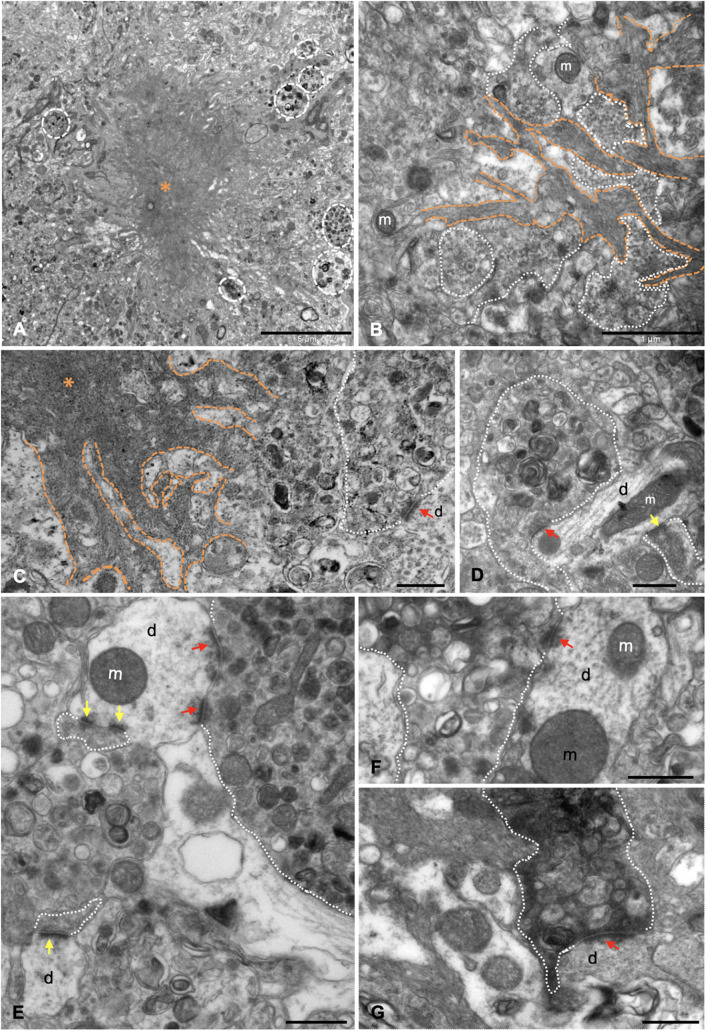
Dystrophic presynaptic boutons, filled with autophagic vesicles, are associated with extracellular Aβ deposits. **(A)** Panoramic view of a neuritic plaque (asterisk) by transmission electron microscopy (TEM), surrounded by dystrophic neurites (discontinuous white circles) in 4.5-month-old APP/PS1. **(B)** Aβ branches (orange discontinuous lines) were found to be intermingled with aberrant neuronal processes (white dotted line); some of them were exhibiting accumulation of small like-synaptic vesicles. **(C–F)** TEM examination demonstrated there were null or very few synaptic contacts around the plaques. In some cases, synapses were established between apparently normal postsynaptic elements and abnormally enlarged presynaptic terminals (red arrows point to postsynaptic densities), filled with vesicles of autophagic nature, and, on the contrary, very few (if any) synaptic vesicles close the presynaptic densities. **(G)** Degenerating synapse (white dotted line) exhibiting an electron dense presynaptic element still keeping contact with a dendritic spine (red arrow). m, mitochondria, d, dendrite/dendritic spine. Yellow arrows point to postsynaptic densities of apparently normal synaptic contacts. Scale bars, **(A)** 5 μm; **(B)** 1 μm; **(C–G)** 0.5 μm.

The presence of dystrophic neurites (axons and presynaptic terminals) surrounding Aβ plaques within the hippocampal formation of this model has been extensively reported (Blanchard et al., 2003; Ramos et al., 2006; Moreno-Gonzalez et al., 2009; Baglietto-Vargas et al., 2010; Sanchez-Varo et al., 2012; Torres et al., 2012; Trujillo-Estrada et al., 2013, 2014; Fernandez-Valenzuela et al., 2020; Sanchez-Mejias et al., 2020). Moreover, the ultrastructural analysis of hippocampus from young (4–6 months of age) APP/PS1 mice revealed the close spatial relationship between amyloid plaques and aberrant neuronal processes (Sanchez-Varo et al., 2012; Gomez-Arboledas et al., 2018). Our ultrastructural analysis indicates that these abnormally swollen neurites displayed a round/oval shape and a giant size (Figure 3A), compared to other normal neuronal processes in the nearby neuropile. Moreover, Aβ fibrils were found to be closely associated with dystrophic neurites, which exhibit vesicular content of autophagic/synaptic nature (Figures 3B,C). In fact, some small presynaptic-like profiles seemed to be mixed with the fibrillar Aβ at the plaque edge. In this sense, our group has previously demonstrated that periplaque aberrant presynaptic terminals are filled with autophagic vesicles containing amyloid precursor protein (APP) and Aβ (Sanchez-Varo et al., 2012). Numerous periplaque SYN-positive dystrophies are found within the hippocampus (Supplementary Figures 1D,F, 2A–C). Double confocal immunofluorescence labeling demonstrated the colocalization of SYN with either the autophagic marker LC3b (Supplementary Figures 2A1–A3) or APP (Supplementary Figures 2B1–B3) within these enlarged structures. The prevalence of SYN, LC3b, or APP was found to be variable among dystrophic neurites (see insets in Supplementary Figures 2A3,B3). Interestingly, SYN colocalized with Aβ42 in swollen neuronal processes both around and within the plaques (Supplementary Figures 2C1–C3). TEM analysis demonstrated the scarce presence of synaptic contacts around the amyloid plaques as soon as 4.5 months of age in APP/PS1 mice. Seemingly, some of them were established between apparently healthy postsynaptic elements and abnormal presynaptic terminals. These elements were filled with many autophagic vesicles and, conversely, very few (if any) synaptic vesicles close to the active zones (Figures 3C–E). Furthermore, some of the presynaptic terminals with vesicular build-up displayed an electron dense cytoplasm (Figures 3F–G), a typical hallmark of degenerating processes. Finally, these terminals were found to establish synaptic contacts with either normal (Figures 3E,F) or abnormal (Figure 3G) postsynaptic components. These periplaque disturbed synaptic boutons may represent the morphological correlate leading to the initial cognitive failure, preceding synaptic and neuronal loss.

### Synaptic Damage Is Proportional to Amyloid-Beta Plaques Closeness and Size

**FIGURE 4:**
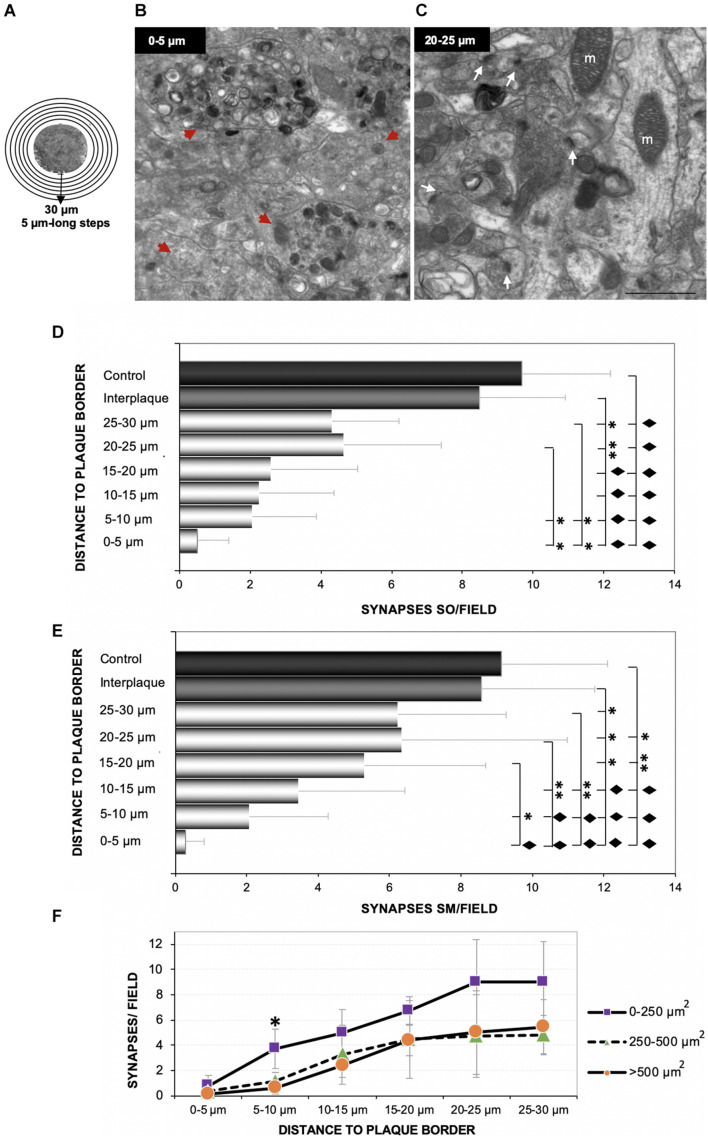
The loss of synaptic contacts increases at closer distance to Aβ plaques. Six consecutive TEM images were taken from several 30-μm-long radii, considering the plaque borders as starting points as shown in panel **(A)**, from 4.5-month-old APP/PS1. Ultramicrophotographs showing the null/scarce number of synapses in areas close to the plaque border [**(B)** 0–5 μm beyond plaque perimeter] compared to further areas [**(C)** 20–25 μm, white arrows] in the stratum moleculare. Moreover, the closer to the plaques, the higher number of dystrophic neurites [red arrowheads in panel **(B)**]. The quantification showed a significant decrease in the amount of synaptic contacts close to the plaque, in comparison to both interplaque spaces either in APP/PS1 mice or age-matched control mice (*n* = 3/group). Synaptic loss inversely correlated with the distance to Aβ plaque in each analyzed layer [**(D)** stratum oriens; **(E)** stratum moleculare]. One-way ANOVA *F*_(7, 21)_ = 35.98; *p* < 0.001, and *F*_(7, 21)_ = 40.81; *p* < 0.001, respectively. Tukey’s ◆*p* < 0.001; ^∗∗^*p* < 0.01; ^∗^*p* < 0.05. **(F)** Finally, we found a relationship between the plaque size and the magnitude of its impact on the nearby synapses [one-way ANOVA *F*_(14, 45)_ = 16.38; *p* < 0.001, ^∗^Tukey’s *p* = 0.027]. m, mitochondria. Scale bars, **(B–C)** 1 μm.

In order to determine the impact of amyloid deposition over synaptic density, we performed an ultrastructural quantification in the hippocampus of 4.5 months APP/PS1 mice. Starting from the edge of the plaques (Figure 4A), six consecutive photomicrographs were taken, covering 3–4 different radii per plaque (30-μm-length radii). Identifiable synaptic contacts (only those displaying clearly visible pre- and post-synaptic elements, and the synaptic cleft) were manually counted within each field. Electron-photomicrographs from interplaque zones and age-matched WT mice were used as control. The analysis showed scarce or none synaptic contacts at the plaque perimeter (Figure 4B, zone between 0 and 5 μm from plaque) in comparison to more distant areas (Figure 4C, 20–25 μm), which were progressively increasing the synaptic density reaching values more similar to control zones. Therefore, we found a significant synaptic loss around the plaques within all the hippocampal regions studied (stratum oriens, Figure 4D; stratum moleculare, Figure 4E; stratum radiatum, not shown). When data were arranged according to plaque size, we observed that the largest amyloid deposits (> 500 μm^2^) provoked a higher synaptotoxic impact, especially close to the plaque border (Figure 4F; one-way ANOVA, *p* = 0.0027). Overall, these data indicated that there is a tight relationship between synaptic damage or loss and extracellular amyloid deposition.

**FIGURE 5:**
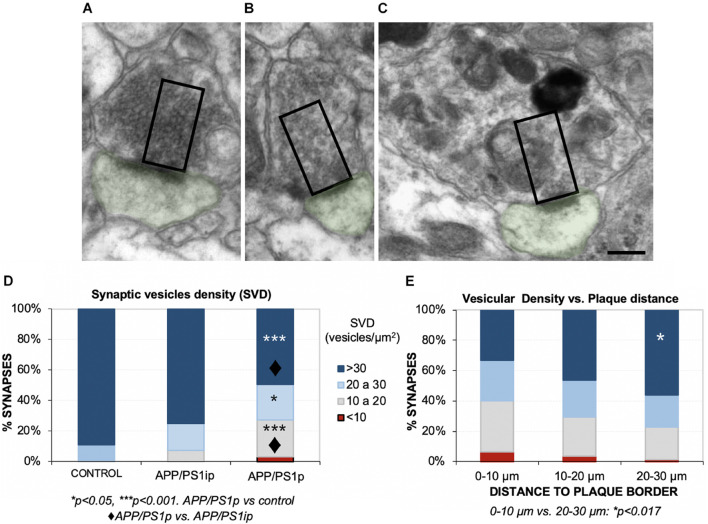
Hippocampal synaptic vesicle density (SVD) decreases in the remaining presynaptic terminals surrounding Aβ deposits. **(A–C)** With the aim of quantifying the SVD at 4.5 months of age, we selected presynaptic terminals with a minimum size (rectangles, 0.5 μm^2^), and manually counted the synaptic vesicles located within that area, close to the postsynaptic density (postsynaptic element in green). Here, some examples of presynaptic terminals with a very high **(A)**, medium-high **(B)**, and low **(C)** vesicular densities are shown. Note that low SVD was usually accompanied by autophagic vesicles build-up, as shown in panel **(C)**. **(D)** Control mice displayed the most elevated ratio of high/intermediate SVDs, which was very similar to the densities from interplaque locations in the double transgenic mice (APP/PS1ip). Periplaque areas (APP/PS1p) exhibited a lower ratio of dense synapses, and synapses with a very low density (<10 vesicles/μm^2^) were detected. Kruskal–Wallis ANOVA, 11 degrees of freedom; *H* = 65.46, *p* < 0.001; Dunn test. *p < 0.05; ***p < 0.001. APP/PS1p vs. control: ◆*p* < 0.001. **(E)** Synapses that were located closer to the plaques displayed less vesicle density. Kruskal–Wallis ANOVA, 11 degrees of freedom; *H* = 27.36, *p* = 0.004; Dunn test: *p < 0.017. Scale bars, **(A–C)** 200 nm.

Next, we focused on the effect of plaques on SVD near to the active zones (releasable pool), a parameter more related with the correct functioning of synapses. To that purpose, we analyzed the SVD in the remaining presynaptic terminals from the same samples. To avoid underestimation of terminals quantification, only presynaptic terminals over 0.5-μm^2^-sized were considered (Parodi et al., 2010). A comparison among several presynaptic elements, showing the counting area, is depicted in Figures 5A–C. The quantification revealed that the periplaque areas (APP/PS1p) displayed a reduced proportion of presynaptic boutons with high vesicular density, compared to the control or interplaque (APP/PS1ip) areas (Figure 5D). Moreover, among APP/PS1p terminals, we found a group of synapses showing a low-density category (<10 vesicles/μm^2^). In addition, the closer to the plaques, the lower SVD within the presynaptic compartments (Figure 5E). Interestingly, the drop in the vesicular density was usually associated with the build-up of autophagic vesicles (Figure 5C). Therefore, these data contribute to shed light on the negative impact of Aβ deposits on the presynaptic function, which was inversely correlated with the distance to plaques.

### The Periphery of Extracellular Deposits Is Enriched in Amyloid-Beta Oligomeric Forms in Both Young APP/PS1 Mice and Human Alzheimer’s Disease Brains

**FIGURE 6:**
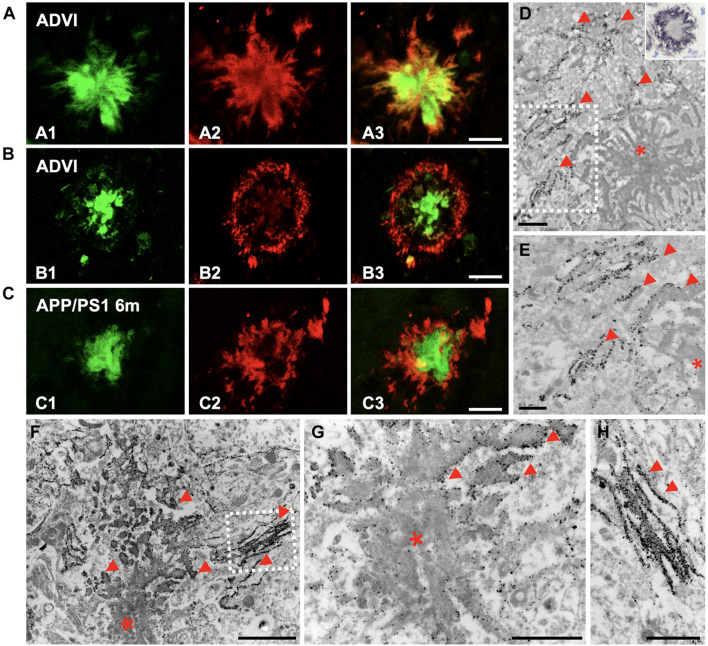
Oligomeric forms of Aβ were located at the plaque periphery in both AD and APP/PS1 hippocampi. **(A,B)** Confocal double-fluorescent staining with Thioflavin-S [**(A1,B1)** green] and OC antibody [**(A2,B2)** red] revealed that oligomeric Aβ was mainly detected at the plaque periphery, depicting a halo around the fibrillar core in CA1 region of Braak VI brains **(A3,B3)**. **(C)** Likewise, OC immunoreactivity showed the same disposition in plaques of 6-month-old APP/PS1 mouse hippocampus **(C1–C3)**. **(D)** Aβ immunogold labeling (OC antibody) observed by TEM was found at the plaque border (red arrowheads; asterisk, Aβ deposit). Detail at higher magnifications in panel **(E)**. Inset in panel **(D)**. Detail of a plaque from CA1, immunostained for amyloid oligomers (OC antibody) in a semithin section from a 6-month-old APP/PS1 mouse. Counterstained with Harry’s hematoxylin. **(F–H)** Immunogold for 12–24 mers Aβ (NU-4) confirmed the presence of these specific amyloid oligomeric forms in the edge and peripheral threads of extracellular Aβ deposits (red arrowheads). Panel **(H)** depict details from panel **(F)**. Scale bars, **(A–C)** 25 μm; **(D,G–H)** 1 μm, **(E)** 0.5 μm; **(F)** 2 μm.

Given that Aβ oligomeric species have been recognized as synaptotoxic agents, we intended to investigate their location within the plaques in the hippocampus from both APP/PS1 mice and AD patients. We performed double-fluorescence staining with Thioflavin-S (ThioS) and the OC antibody in human AD samples (Braak VI stage), showing that the oligomeric Aβ was located at the plaque edge, surrounding the fibrillar core (Figures 6A,B). Interestingly, the plaques from 6-month-old APP/PS1 mice exhibited the same OC/Thio-S relative distribution as human deposits did (Figure 6C). In addition, we performed OC immunohistochemistry on semithin sections from APP/PS1 hippocampus, which was mainly detected at the plaque periphery (detail at higher magnification of CA1 deposit in inset on Figure 6D). The ultrastructural analysis of Aβ immunogold-labeled sections using two different conformational antibodies (Figures 6D,E, OC antibody; Figures 6F–H, NU-4 antibody) corroborated the location of these specific Aβ species at the plaque periphery, bordering the Aβ fibrils. All these data demonstrated the preferential disposition of Aβ oligomers in the periphery of extracellular plaques.

**FIGURE 7:**
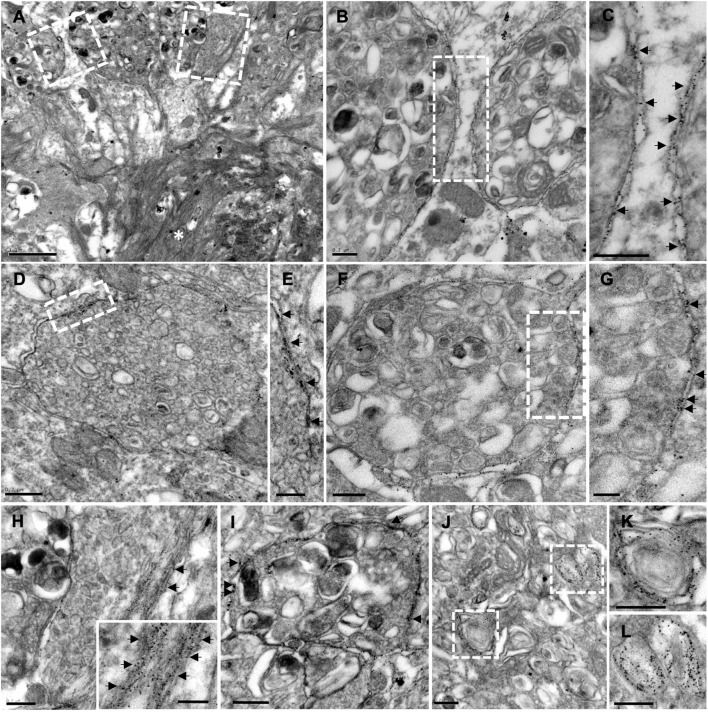
Plaque-associated dystrophic neurites as a source of Aβ42. TEM images from Aβ42-immunogold labeled hippocampus from 6-month-old APP/PS1 mice. **(A)** Aβ42-labeling evidenced the presence of this amyloid isoform, not only within the plaque (asterisk), but also in the membrane of periplaque dystrophic neurites **(B–H)**. Arrows in the corresponding details **(C,E,G)** and **(H,I)** point to the gold particles. Inset in panel **(H)** depicts amyloid fibers intermingled with a dystrophic process. **(I,J)** Aβ42-positive swollen axonal/presynaptic neuronal processes were filled with vesicular compartments related to autophagy-lysosomal pathway [details at higher magnification in panels **(K,L)**], which were positive to Aβ42. Scale bars, **(A)** 1 μm; **(B–D,F,H–L)** 0.2 μm; **(E–G)**, inset in panel **(H)** 0.1 μm.

Importantly, immunogold staining in 6-month-old APP/PS1 mice evidenced the presence of Aβ42 isoform, not only in the plaques, but also in the membrane of periplaque dystrophic neurites (Figures 7A–H; the inset in H depicts amyloid fibers intermingled with a dystrophic process). These Aβ42-positive swollen axonal/presynaptic neuronal processes were filled with vesicular compartments related to autophagy-lysosomal pathway (Figures 7I–L) as previously described in this model hippocampus, supporting the idea of dystrophic neurites as a place of APP processing, amyloid generation, and release (Sanchez-Varo et al., 2012).

### Soluble Fractions From Hippocampus of Young APP/PS1 Mice Induce Synaptic Damage in Primary Neuronal Cultures

**FIGURE 8:**
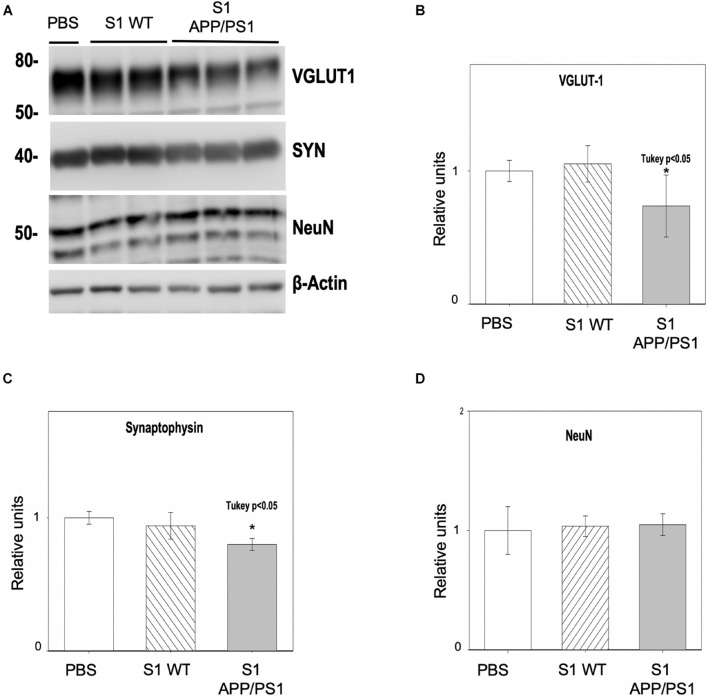
Hippocampal soluble fractions from young APP/PS1 mice reduce the synaptic protein content *in vitro*. Primary neuronal cultures were incubated with soluble fractions (S1) from 6-month-old APP/PS1 or WT mice for 48 h (*n* = 3/group). Incubation with PBS was used as an additional control. Analysis by Western blot **(A)** and quantification showed a significant decrease in the protein levels of the presynaptic markers VGLUT1 **(B)** [ANOVA *F*_(2, 9)_ = 4.38 *P* = 0.05; Tukey’s *p < 0.5] and SYN **(C)** [ANOVA *F*_(2, 9)_ = 8.03 *P* = 0.05; Tukey’s *p < 0.05] after incubating with APP/PS1 S1, but not when incubating with control fractions. As a proof of the selective synaptic damage, NeuN protein levels showed no significant changes **(D)**. Represented data were normalized respect to PBS.

To confirm the presence of soluble synaptotoxic molecules in APP/PS1 mice at early ages, primary neuronal cultures were incubated with soluble fractions (S1) from 6-month-old APP/PS1 hippocampus for 48 h. S1 from age-matched WT mice or PBS were used as controls. The quantitative analysis (Figure 8A) demonstrated that the levels of the presynaptic markers VGLUT1 (Figure 8B) and SYN (Figure 8C) were significantly decreased (−26.29 ± 23.26% and −20 ± 4.5%, respectively), after incubating cells with S1 from APP/PS1 hippocampus, but not in PBS or WT S1 fractions. As supporting evidence of specific synaptic protein loss, we analyzed the neuronal marker NeuN (Figure 8D) which remained unchanged (4.84 ± 9.13%), and rule out neuronal death as a direct cause of this synaptic drop.

## Discussion

Over the last three decades, the relationship between the loss of synapses and cognitive impairment in AD has been progressively acknowledged (Terry et al., 1991; Selkoe, 2002; Scheff et al., 2006, 2007; Shankar and Walsh, 2009; Spires-Jones and Hyman, 2014; Forner et al., 2017; John and Reddy, 2021). NFTs are known to strongly correlate with cognitive dysfunction, and synaptic and neuronal degeneration. However, the role of amyloid plaques in AD synaptopathology has been a subject of deep controversy. Some studies established a correlation between plaques and cognitive failure in the early stages of the disease, especially with neuritic plaques (Nelson et al., 2007; for review Nelson et al., 2012). In the present study, we described an early spatial memory decline accompanied by synaptic pathology, which was associated to extracellular oligomeric/fibrillar Aβ in the hippocampus of the APP/PS1 mice. Importantly, synaptic damage was not only circumscribed to the loss of synaptic contacts, but also involved the decrease of SVD. Therefore, presynaptic vesicular depletion might represent one of the first pathological indicators and a physiological substrate of early synaptic/cognitive dysfunction. Thus, dysfunctional presynaptic elements may be considered a relevant therapeutic target, especially to the prodromal and early stages of this neurodegenerative disease. Therefore, synaptic/cognitive evaluation of transgenic AD models represents an indispensable step in their characterization to obtain reliable preclinical tools for testing potential synaptoprotective interventions.

In this work, we have demonstrated an early plaque-associated synaptic pathology in the hippocampus of an APP/PS1 model by combining molecular, immunohistochemical, and ultrastructural approaches. Previously, we and others had reported that amyloidogenic transgenic mice reproduce the formation of neuritic plaques. This type of amyloid deposits is accompanied by synaptic/axonal dystrophies with accumulation of multiple cellular components. Specifically, the build-up of autophagic/lysosomal vesicles, ubiquitin and phospho-tau, together with the presence of synaptic proteins such as SYN, and markers of specific glutamatergic (VGLUT), GABAergic (VGAT, SOM, NPY, and CR), and cholinergic (ChAT) neuronal subpopulations have been described in this model (Blanchard et al., 2003; Delatour et al., 2004; Ramos et al., 2006; Moreno-Gonzalez et al., 2009; Baglietto-Vargas et al., 2010; Sanchez-Varo et al., 2012; Torres et al., 2012; Trujillo-Estrada et al., 2013, 2014; Gomez-Arboledas et al., 2018; Fernandez-Valenzuela et al., 2020; Sanchez-Mejias et al., 2020). The synaptic loss takes place not only within the plaque core but also in the close proximity. Initially, the direct plaque impact over neuropil was assessed by light microscopy immunostainings, evidenced by quantifying immunonegative areas (plaque effect as a physical entity) of the ubiquitous presynaptic protein SYN, and finally corroborated by TEM (covering 30-μm beyond plaque perimeter). SYN is a general vesicular presynaptic protein, relevant for cognitive processes, synaptic plasticity, SV endocytosis, and recycling (Schmitt et al., 2009; Kwon and Chapman, 2011). According to our data, the loss of SYN and other synaptic proteins has been extensively reported in AD patients (Masliah et al., 1989, 1990, 2001; DeKosky and Scheff, 1990; Terry et al., 1991; Scheff and Price, 2003; Yao et al., 2003; Ishibashi et al., 2006; Shinohara et al., 2014; de Wilde et al., 2016; Kurucu et al., 2021) and mouse models (Games et al., 1995; Mucke et al., 2000; Rutten et al., 2005; Spires et al., 2005; Saito et al., 2014; Sasaguri et al., 2017; for review, John and Reddy, 2021). For instance, Mitew et al. (2013) showed that the relative presynaptic bouton density containing SYN and VGLUT1 was extremely affected in 12-month-old APPswe/PS1 mice, in both plaque areas and its periphery. Furthermore, Aβ deleterious effect on the synaptic density was directly correlated with the plaque proximity. Similar findings have been reported in the Tg2576 mice in both stratum molecular and entorhinal cortex, though especially at older ages (Dong et al., 2007). In this case, the drop in the synapse number was significant until 200-μm far from plaques. Such a difference might be explained as the plaque number and size is usually age-dependent. In fact, the greatest differences were found in those mice that were significantly older (15- to 18-month-old vs. 6- month-old) in comparison to our model (4.5–6 months). Kirkwood et al. (2013) described alterations and loss of dendritic spines in the PSAPP model very near plaques (6 μm beyond the perimeter). Interestingly, Koffie et al. (2009) showed a progressive loss of PSD95-immunopositive puncta (60%), reaching up to 50 μm from plaque edge by using array tomography in the somatosensory cortex of old APP/PS1Delta9 mice (8–13 months). In addition, Robinson et al. (2014) described a relationship between dementia and SYN loss in the perforant pathway, in which hippocampus is specially involved. In this sense, our study essentially covers different hippocampal layers implicated in the trisynaptic circuit. Additionally, since we have previously reported that there is no change in terms of SYN expression in this model hippocampus (at 6 months of age) (Ramos et al., 2006), SYN protein level reduction must be happening at the post-transcriptional level.

In the last decades, compelling evidence have highlighted the role of soluble oligomers as the most toxic forms of Aβ, posing the oligomer hypothesis (for review, see Cline et al., 2018). These pathogenic oligomeric species are able to interfere directly with synaptic functions, correlating better than plaques with cognitive dysfunction in AD (Lambert et al., 1998; McLean et al., 1999; Wang et al., 1999; Baglietto-Vargas et al., 2018; Cline et al., 2018; Li and Selkoe, 2020). In fact, the concentration of soluble Aβ was shown as a relevant correlate to discern between AD patients and pathological controls without dementia or synapse loss (Lue et al., 1999). Oligomers have been described to be initially formed within neuronal dystrophic processes and synapses (Takahashi et al., 2002, 2004; Sanchez-Varo et al., 2012). The presence of APP, Aβ, and secretases within presynaptic compartments supports this hypothesis (Sanchez-Varo et al., 2012; Torres et al., 2012; Sadleir et al., 2016). There is extensive evidence obtained from AD brains (Plowey and Wiley, 2007; Nixon et al., 2008; Tramutola et al., 2015) and models (Majumder et al., 2011; Steele et al., 2013; Yang et al., 2008, 2011), indicating that endolysosomal autophagy deficiencies play a role in AD and amyloidogenesis (for review see Nixon, 2017; Cao et al., 2019). Therefore, restoring autophagic function is considered another therapeutical target for this disease (Friedman et al., 2014; Wheeler et al., 2015). APP/PS1 mice reproduce the accumulation of these type of vesicles in presynaptic dystrophies as well (Sanchez-Varo et al., 2012; Torres et al., 2012). In this work, both confocal double staining and TEM revealed different ratios of synaptic/autophagic vesicle build-up within abnormally swollen presynaptic compartments, which are immunopositive for Aβ42. These facts were consistent with our previous report (Sanchez-Varo et al., 2012), depicting autophagic vesicles gold-labeled for APP and Aβ42 inside of morphologically disrupted neuronal processes, supporting that autophagic failure participates in Aβ secretion/accumulation (Yu et al., 2005; Nilsson and Saido, 2014).

Some groups have reported that SYN levels decreased *in vitro* when incubating with Aβ oligomers (Peters et al., 2015; Bate and Williams, 2018). Importantly, in addition to intraneuronal Aβ, plaques have been proposed as potentially major sources of soluble and toxic oligomeric Aβ (Martins et al., 2008), and the proximity of plaques facilitates an Aβ-induced excitotoxic cascade (McLellan et al., 2003). In this sense, we have found a special enrichment of several oligomeric forms in the plaque perimeter of both APP/PS1 and AD hippocampal samples. We have also shown that soluble S1 fraction from APP/PS1 hippocampus, which is enriched in oligomeric amyloid-β (Jimenez et al., 2011; Sanchez-Varo et al., 2012; Sanchez-Mico et al., 2021), exhibits synaptotoxic potential *in vitro*. Altogether, these data may account for the synaptic damage/loss observed near the hippocampal extracellular deposits.

In this same line, we have demonstrated not only a decrease in the density of synapses related to the periplaque oligomeric halo but also a specific damage in terms of efficiency in the remaining synaptic boutons, in the form of a lower SVD through several hippocampal strata. Correspondingly, Parodi et al. (2010) reported a decrease in the SV pool *in vitro* after incubating hippocampal neurons for 24 h with different subsets of Aβ oligomers. Moreover, it has been proposed that Aβ oligomers interact with the neuronal membrane, inducing perforations (Sepulveda et al., 2010). Consequently, calcium ions entry and a large release of SVs take place, leading to a delayed synaptic failure by presynaptic vesicle depletion (Parodi et al., 2010; Sepulveda et al., 2010; Peters et al., 2013, 2015). There are evidences pointing to the involvement of the cellular prion protein PrPc in this mechanism, among other molecules (Laurén et al., 2009; Peters et al., 2015; Bate and Williams, 2018; Li and Selkoe, 2020). It is also generally accepted that oligomers induce the overstimulation of NMDA receptors, triggering a pathological cascade of events leading to synaptic dysfunction/disruption (for review, Cline et al., 2018). Our previous data suggest that SV pool in this model might be affected by Aβ-induced failures in cytoskeleton (actin-cofilin rods), altered trafficking, and autophagy within dystrophic axons and presynaptic compartments (Sanchez-Varo et al., 2012; Torres et al., 2012). Furthermore, the reduction in the vesicular pool might be due to an inappropriate performance of the SV endocytosis as well. Importantly, synaptic function and, specifically, SV exocytosis have been linked with neuronal Aβ release (Cirrito et al., 2005, 2008; Bero et al., 2011). In fact, picomolar Aβ concentration enhances synaptic plasticity and memory (Puzzo et al., 2008; for review, Kent et al., 2020). Additionally, monomers have been described to exert a neuroprotective role (Brody et al., 2008; Giuffrida et al., 2009; Bate and Williams, 2018). Thus, endogenous Aβ peptides are suggested to have a crucial role in activity-dependent regulation of SV release, and consequently, amyloid alterations might point to the primary pathological events that lead to synapse alterations in AD (Abramov et al., 2009). Moreover, it has been shown that synaptic activation promotes amyloid secretion, whereas chronic reduction of synaptic activity reduces plaque loading in an AD transgenic mouse model (Cirrito et al., 2005, 2008; Tampellini et al., 2010).

Certainly, the disappearance of synapses involves the loss of both pre- and post-synaptic compartments. Here, we have reported a significant decrease of hippocampal PSD95 protein levels in the APP/PS1 mouse, together with the nearly absence of dendrites (MAP2) in plaque locations, as we and others have shown (Boutajangout et al., 2004; Sanchez-Varo et al., 2012). Moreover, we have found that periplaque aberrant presynaptic terminals were generally associated to apparently normal postsynaptic compartments (dendritic shafts and spines), and sometimes with degenerating spines. Thus, our ultrastructural analysis suggests that presynaptic damage may precede some spine alterations, although these postsynaptic compartments have not been specifically assessed in detail along the present work. Indeed, the diffusion of soluble synaptotoxic oligomers between both the pre- and post-synaptic elements is postulated as a mechanism of pathological spreading (Spires-Jones and Hyman, 2014). Even though we have not found Aβ within postsynaptic compartments at this early age, we cannot rule out this possibility. Furthermore, Aβ has been also considered as a post-synaptic negative regulator at higher concentrations (Palop and Mucke, 2010), and an association between Aβ and PSD95 levels has been previously demonstrated (Koffie et al., 2009, 2011; Shinohara et al., 2013, 2014; Baglietto-Vargas et al., 2018). In this regard, postsynaptic alterations in hippocampal culture developed after a prolonged exposure to higher Aβ concentrations than those needed for the presynaptic deficits (Parodi et al., 2010). Other authors have shown that spine density around amyloid plaques reduces after some weeks of plaque formation, and later than presynaptic damage (Bittner et al., 2010, 2012; Dorostkar et al., 2015). Overall, these results support that alterations within the presynaptic compartment may occur earlier than in the postsynaptic site, and before synapse loss in the pathological course of AD (de Wilde et al., 2016). Therefore, our observations suggest that presynaptic affectation may constitute the first step in synaptic extinction, at least in this AD model. Certainly, Aβ control of synaptic function seems to require a very tight regulation and depends on the β-amyloid conformation and concentration. Finally, we have detected a decrease in PSD95 expression in pyramidal cells from APP/PS1 mice between 4 and 6 months of age (data not shown), suggesting the existence of other mechanisms leading to the progressive alteration of post-synaptic efficiency in parallel to the drop of synaptic vesicles. On the whole, these data point to the Aβ loss/alteration-of-function as another possible cause of AD neuropathogenesis.

To conclude, there could coexist different mechanisms involved in AD-synaptic loss. It is highly likely that synaptic vesicles and boutons loss is a consequence of a synergic effect, between extracellular Aβ effect and a collapse in axonal/presynaptic transport. This transport impairment may be due to autophagic and cytoskeletal disturbances (Sanchez-Varo et al., 2012; Torres et al., 2012; Fernandez-Valenzuela et al., 2020), in which tau pathology is also clearly involved (Spires-Jones and Hyman, 2014; John and Reddy, 2021). Importantly, reactive astrocytes have been reported to enwrap periplaque presynaptic dystrophies in this model and AD samples (Gomez-Arboledas et al., 2018). However, Aβ itself, but not tau, progressively impairs the capacity of these glial cells to eliminate damaged amyloid-containing presynaptic elements (Sanchez-Mico et al., 2021). Besides, microglial cells are implicated in synaptic homeostasis as well, and the rate of maintenance/elimination of boutons or perineuronal nets might be altered due to amyloid exposition (Hong et al., 2016; Crapser et al., 2020, 2021). Soluble Aβ itself can induce synaptic alterations in the absence of plaques (Lue et al., 1999; Dorostkar et al., 2015). Indeed, Aβ oligomeric species are sufficient to trigger alterations in synaptic plasticity, synaptic loss, neuroinflammation, memory deficits, and neurodegeneration, in the absence of plaques and/or combination with other phospho-tau downstream pathological pathways (Cline et al., 2018; Li and Selkoe, 2020; John and Reddy, 2021), but this subject goes beyond the present work. The plastic nature of synapses, together with the undeniable implication of synaptic loss in AD-related cognitive deficits, points at these structures as attractive targets for early therapeutic interventions. Indeed, the drop of functionality may be considered a relevant step prior to synaptic degeneration, leading to abnormal communications among neurons, and eventually to cognitive impairment. Finally, animal models exhibiting early oligomeric-associated synaptopathology constitute relevant tools for testing novel and meaningful pharmacological treatments against this devastating disease.

## Data Availability Statement

The original contributions presented in the study are included in the article/Supplementary Material, further inquiries can be directed to the corresponding authors.

## Ethics Statement

The animal study was reviewed and approved by the biobank and the Animal Research Committee from the University of Malaga and Seville (Spain) following Spanish legislation. The patients/participants provided their written informed consent to participate in this study.

## Author Contributions

RS-V, AG-A, and JV conceived the study, analyzed and interpreted the data, and wrote the manuscript. RS-V, ES-M, LT-E, and MM-O performed the histology and immunohistochemical experiments and the image analysis quantifications. RS-V, JD, and AG-A performed the transmission electron microscopy experiments and quantifications. VC and JF-V performed the behavioral studies and contributed to the data analysis. SJ and MS-M performed the cell culture and molecular studies. RS-V, ES-M, LT-E, and SJ designed the figures. MV, DB-V, and IM-G critically revised the manuscript for intellectual content. All authors read and approved the final manuscript before submission.

## Conflict of Interest

The authors declare that the research was conducted in the absence of any commercial or financial relationships that could be construed as a potential conflict of interest.

## Publisher’s Note

All claims expressed in this article are solely those of the authors and do not necessarily represent those of their affiliated organizations, or those of the publisher, the editors and the reviewers. Any product that may be evaluated in this article, or claim that may be made by its manufacturer, is not guaranteed or endorsed by the publisher.
