# Supplementary material for: Plaque-Associated Oligomeric Amyloid-Beta Drives Early Synaptotoxicity in APP/PS1 Mice Hippocampus: Ultrastructural Pathology Analysis
Source: Front Neurosci. 2021 Nov 4;15:752594. doi: 10.3389/fnins.2021.752594 (PMC8600261; doi:10.3389/fnins.2021.752594)
Supplement: Supplementary file 1 [file Data_Sheet_1.PDF]

Supplementary Material

Table 1. Demographic data for *post-mortem* human brains

Case	Age	Gender	*Post-mortem* delay	Braak stage	CERAD stage	Death cause
Case 1	56	Female	7h	V-VI	C	Bronchoaspiration
Case 2	63	Female	13h 50m	V-VI	B	Pulmonary neoplasia
Case 3	77	Female	16h 30m	V-VI	C	-
Case 4	93	Male	5h	V-VI	C	-
Case 5	76	Male	6h	V-VI	B	-

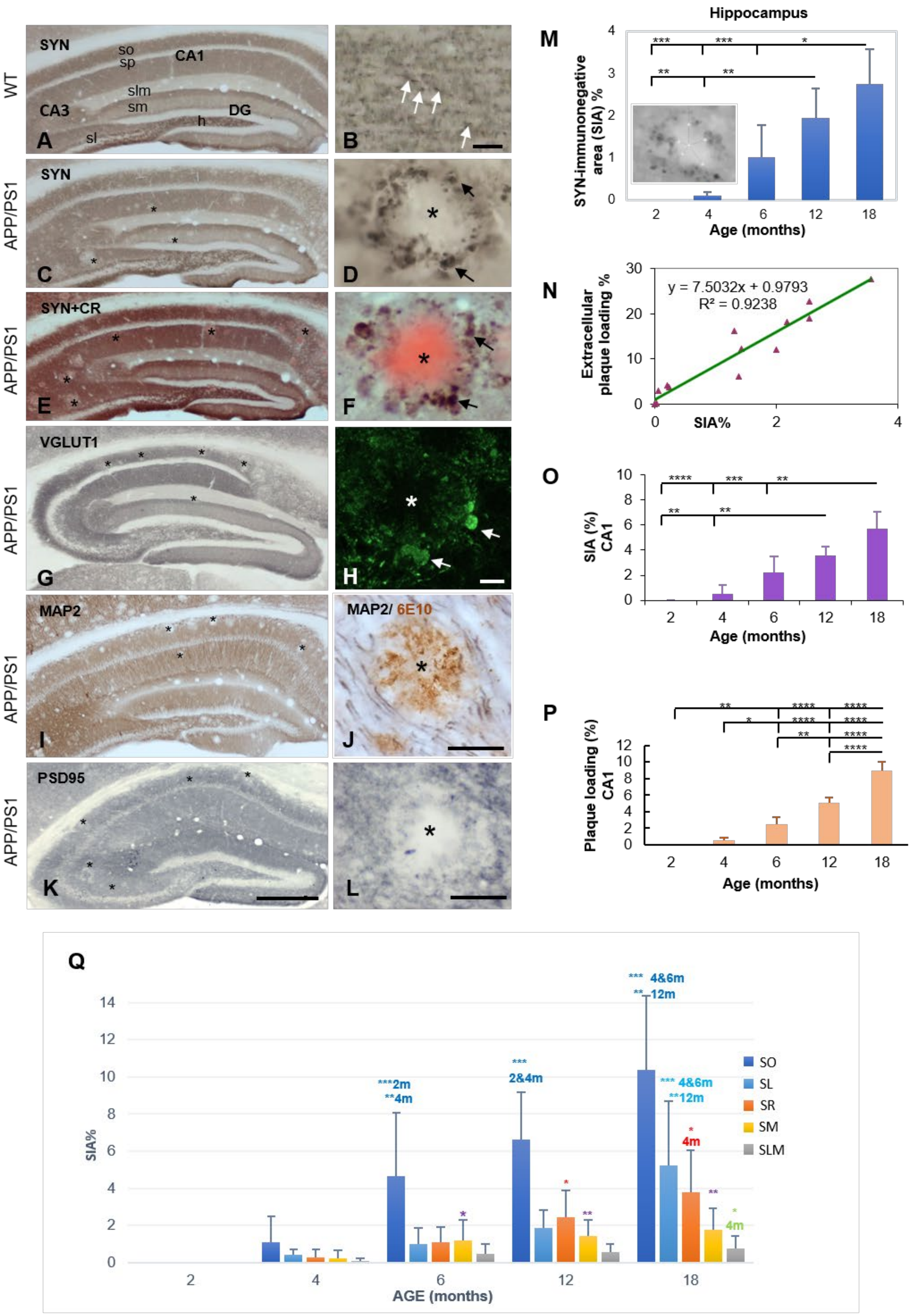

St. Oriens >> St. Lucidum >St. Radiatum >St. Moleculare >St. Lacunosum Moleculare

*SO: One-way ANOVA F(4,40) = 33.67 p<0.001. SR and SLM: One-way ANOVA F(4,40)= 7.822 and 4.81 respectively. p=0.003. SL: One-way ANOVA F(4,40)= 12.24 p<0.001. SM: One-way ANOVA F(4,40)= 7.77 p<0.001. Tukey test ***p<0.001; **p<0.01; *p<0.05. 2m vs. 18m ***p<0.001.*

Supplementary Figure 1. The loss of synaptic and dendritic markers immunoreactivity in APP/PS1 hippocampus is associated to amyloid deposition.

Immunohistochemical stainings for the presynaptic proteins SYN (**A, C**) and VGLUT1 (**G**) exhibited their typical laminar pattern in the hippocampus (6 months). (**C-H**) From early ages, APP/PS1 presynaptic terminals appeared directly affected by the presence of amyloid plaques (**E-F**, double staining SYN/Congo red (CR); **G-H,** glutamatergic terminals (VGLUT1); asterisks indicate plaque location). (**B, D, F**) Compared to free-of-plaque regions (**B**, detail; white arrows point to SYN-positive puncta), SYN-immunoreactivity disappeared in the presence of Aβ deposits (**D, F**). (**G-H**) VGLUT1 immunohistochemistry evidenced this effect over glutamatergic presynaptic terminals as well. SYN and VGLUT1 accumulated within periplaque dystrophic neurites (arrows in **D**, **F and H**). Similarly, MAP2 (**I-J**) and PSD95 (**K-L**) showed the lack of dendrites and spines (respectively) within plaque locations (asterisks). **(J)** Detail of double staining MAP2/6E10 in 6-month-old APP/PS1 mice evidence plaque impact over dendrites. (**M**) The percentage of SYN-immunonegative area (SIA) in the hippocampus of APP/PS1 mice was measured with the nucleator method (see inset) from 2 to 18 months of age (0.1% at 4 months, 1% at 6 months ($p=0.06$ vs. 2m); 1.95% at 12 months (2m, 4m vs. 12m **$p<0.01$), 2.75% at 18 months (2-4m vs. 18m ***$p<0.001$; 6m vs. 18m *$p<0.05$). The quantification revealed a significant age-related decrease of SYN-staining (One-way ANOVA $p<0.001$. Tukey test ***$p<0.001$; **$p<0.01$; *$p<0.05$). (**N**) As expected, estimated regression line demonstrated a significant correlation between SIA% and plaque-loading ($R^2 = 0.9238$; $R = 0.96$). (**O**) SIA quantification showing the age-related progression in CA1 region (One-way ANOVA $p<0.001$. Tukey test ***$p<0.01$; **$p<0.01$; *$p<0.05$; 2m vs. 6m $p=0.06$). **(P)** Plaque loading of CA1 region evidence the age-dependent progression pattern of amyloid deposition (6E10 monoclonal antibody), in parallel to SIA. One-way ANOVA $F(4,15) = 91.34$ $P<0.0001$. Tukey test ****$p<0.0001$; ***$p<0.01$; **$p<0.01$; *$p<0.05$. (**Q**) SIA% were independently determined in several hippocampal sublayers, displaying the progressive age-associated loss of SYN (CA1 SO: One-way ANOVA $F(4,40) = 33.67$ $p<0.001$. CA1 SR and SLM: One-way ANOVA $F(4,40)= 7.822$ and 4.81 respectively, $p=0.003$. CA3 SL: One-way ANOVA $F(4,40)= 12.24$ $p<0.001$. DG SM: One-way ANOVA $F(4,40)= 7.77$ $p<0.001$. Tukey test ***$p<0.001$; **$p<0.01$; *$p<0.05$; 2m vs. 18m ***$p<0.001$). So: stratum oriens; sp: stratum pyramidale; slm: stratum lacunosum moleculare; sm: stratum moleculare; sl: stratum lucidum; h: hilus; DG: dentate gyrus. Scale bars, A, C, E, G, I, K 500 μm; B 4 μm; D, F, H 10 μm; J, L 20 μm.

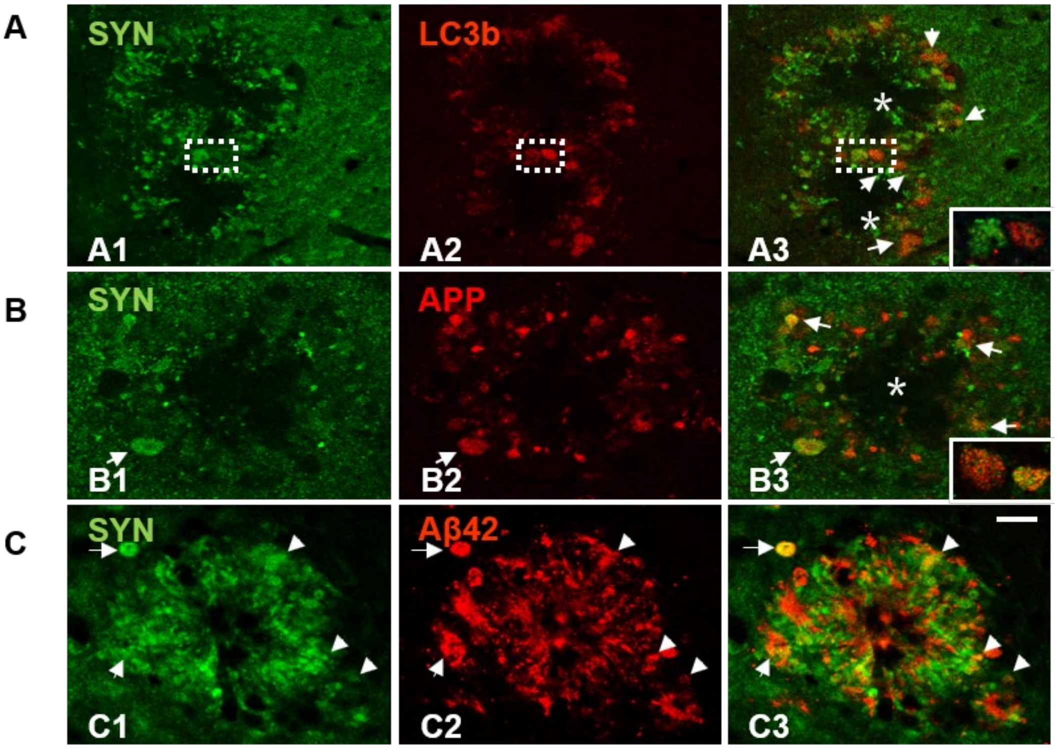

Supplementary Figure 2. SYN-positive dystrophic neurites are associated with Aβ deposition in the APP/PS1 hippocampus.

(A-B-C) Confocal double immunolabeling demonstrated the colocalization of SYN with other different proteins within dystrophic neurites around plaques (asterisks) of 4/6-month-old APP/PS1 mice. (**A1-A3**) These dystrophies stained positively with either anti-SYN (A1, green) or anti-LC3b (A2, red) antibodies, but also for both markers simultaneously at different ratios (white arrows; see detail of hypertrophied neuronal processes in A3 inset). (**B1-B3**) The same situation was found with the double immunostaining for SYN (B1, green) and APP (B2, red) within periplaque dystrophies. See inset in B3 for higher magnifications. (**C1-C3**) SYN (C1, green) and Aβ42 (C2, red) threads revealed to be tightly associated. Moreover, SYN was also found to colocalize with Aβ42 in some dystrophic neuronal processes (white arrows, C3). Scale bars, 10 µm.
